# Minimum Data Set and Metadata for Active Vaccine Safety Surveillance: Systematic Review

**DOI:** 10.2196/63161

**Published:** 2025-06-17

**Authors:** Mengdi Zhang, Junting Yang, Yan Li, Yuan Li, Tong Li, Ziqi Dong, Shuo Gong, Yahui Wu, Minrui Ren, Chunxiang Fan, Lina Zhang, Yi Wang, Yali Wang, Jingtian Ren, Feng Sun, Chuanyong Shen, Keli Li, Zhike Liu, Siyan Zhan

**Affiliations:** 1 Department of Epidemiology and Biostatistics, School of Public Health, Peking University, 38 Xueyuan Road, Haidian District, Beijing, 100191, China, 86 10-82805162, 86 10-82805162; 2 Key Laboratory of Epidemiology of Major Diseases, Peking University, Ministry of Education, Beijing, China; 3 National Immunization Program, Chinese Center for Disease Control and Prevention, Beijing, China; 4 Center for Drug Reevaluation, National center for ADR Monitoring, Beijing, China; 5 Center for Intelligent Public Health, Institute for Artificial Intelligence, Peking University, Beijing, China; 6 Research Center of Clinical Epidemiology, Peking University Third Hospital, Beijing, China

**Keywords:** vaccine safety, active surveillance, minimum data sets, metadata, association

## Abstract

**Background:**

Active vaccine safety surveillance (AVSS) stands as a top priority for the World Health Organization (WHO), serving as a critical indicator of the fourth maturity level for national regulatory agencies.

**Objective:**

This review aims to define the minimal data scope for association studies in vaccine safety, providing a reference framework for implementing AVSS systems worldwide, especially in low- and middle-income countries.

**Methods:**

The study systematically searched PubMed, Embase, and Web of Science for cohort and case-control studies related to AVSS published between January 1, 2018, and September 7, 2022. Guided by the WHO and Council for International Organizations of Medical Sciences guidelines (CIOMS), we developed a 4D framework for Minimum Data Sets (MDSs), including “Vaccine,” “Outcome,” “Demographic Data,” and “Covariate.” Variables with a frequency of at least 5% were included in the MDS.

**Results:**

Of the 123 included studies, 102 (82.9%) were cohort studies and 98 (79.7%) originated from high-income countries, covering populations across the entire life course. The MDS for COVID-19 vaccines identified 54 variables, while the MDS for maternal populations included 96 variables. WHO guidelines were found to align better with practical applications compared to CIOMS guidelines, though both require further optimization based on the MDS findings. However, metadata for these essential variables were inadequately described across the studies.

**Conclusions:**

The proposed MDS provides clear guidance and concise requirements for AVSS data scope. Establishing a globally standardized MDS and comprehensive metadata based on these findings is essential to enhancing the global vaccine safety ecosystem.

## Introduction

Vaccines are one of the most cost-effective interventions for preventing and controlling infectious diseases, and vaccination could reduce 2‐3 million deaths annually, with an additional 1.5 million deaths that could be averted if the coverage expanded [1]. However, because vaccines are administered to healthy individuals, concerns about their safety can significantly impact public trust, leading to vaccine hesitancy. This hesitancy poses serious threats to global health, such as the resurgence of nearly eradicated diseases and challenges in achieving the Immunization Agenda 2030 (IA2030) goals [2]. To address these challenges, robust systems for monitoring vaccine safety are essential. These systems must quickly and accurately evaluate vaccine risks and benefits while providing timely responses to safety concerns, which not only strengthens public confidence but also facilitates effective communication during vaccine rollouts [3,4]. Regulatory frameworks have increasingly recognized the importance of such measures. For instance, the US Food and Drug Administration (FDA) has mandated life-cycle safety management for vaccines since 2007, while China’s Vaccine Administration Law enforces stringent safety requirements from development through postmarket surveillance [5,6]. From the perspective of evidence and monitoring approaches, the premarket clinical trials and postmarket surveillance should be essential measurements to ensure vaccine safety. While clinical trials are critical for assessing safety and efficacy before approval, proactive monitoring systems are indispensable for identifying rare or unforeseen safety issues that may not emerge during trials [7]. Active surveillance enables the timely detection of potential safety signals and provides robust evidence of vaccine benefits, complementing the limitations of passive surveillance [8].

Over the last decade, an increasing number of countries, particularly those in resource-rich regions, are attaching great importance to and accelerating the progress of active vaccine safety surveillance (AVSS) systems [9,10]. Several exemplary models were established and provided high-quality evidence for the critical decision-making of safety in immunization programs [11-16]. There are three types of data collection approaches and different functions among them. First, the Vaccine Safety Datalink (VSD) project collaborated with the Centers for Disease Control and Prevention in the United States and integrated a whole life-course cohort through data linkage, which covered 18 million individuals and included pregnant women, infants, children, and older people [17,18]. Second, the national pediatric hospital surveillance networks were established to enhance routine capacity and bridge the research gap to measure the impact and safety of vaccine programs [19]. Third, AusVaxSafety actively collects adverse events from national immunization sentinel sites through SMS text messaging or email reports [20]. In brief, these systems have gone through many years of investment and construction and now are stable, sustainable, and fast-response due to their large-scale and representative sample size.

The World Health Organization (WHO) has recently been urging and endeavoring to establish a global vaccine safety surveillance ecosystem that highlights the capacity of proactive vigilance in vaccine safety [21]. However, few studies were from low- and middle-income countries (LMICs) due to challenges in securing the necessary funding, technology, and human resources for these systems.

The use of electronic health data for AVSS requires addressing three key questions: (1) What information will researchers and policymakers need in the future for AVSS? (2) What data sources can identify this information? (3) What are the minimum data elements required? The Minimum Data Set (MDS) is a structured collection of essential data elements used for clinical and research purposes, facilitating standardized and efficient data collection. Many countries have adopted MDS frameworks to ensure data comparability, enhance public health decision-making, and support real-time safety monitoring [22]. In the context of AVSS, a well-defined MDS can help identify critical surveillance indicators, ensure data accessibility, and improve risk assessment methodologies [23]. Furthermore, metadata, which provides descriptive information about datasets—plays a key role in standardizing data classification, integration, and analysis across different surveillance systems [24]. Therefore, establishing a standardized MDS and metadata system is essential for optimizing AVSS, particularly in resource-limited settings.

Currently, 2 guidelines provide frameworks for data elements, approaches, and study designs of AVSS, but inconsistencies between these guidelines create challenges in standardizing data collection and usage [25,26]. Furthermore, no study has systematically evaluated or validated the essential data elements proposed by these frameworks, leaving their relevance and applicability uncertain. Robust AVSS systems must rely on credible and essential data to function effectively. To address this gap, our systematic review examines the data elements and metadata requirements for AVSS by synthesizing findings from association studies in vaccine safety. This review aims to define an MDS that ensures efficient AVSS implementation, particularly in resource-limited settings [27].

## Methods

### Search Strategy and Selection Criteria

We systematically searched 3 databases, PubMed, Embase, and Web of Science, from January 1, 2018, to September 7, 2022, to identify all the cohort studies and case-control studies of AVSS. The search strategy was registered on the PROSPERO (International Prospective Register of Systematic Reviews) database (CRD42023449920). The details are shown in Supplementary Materials (Multimedia Appendix 1).

There were two stages in the literature screening. In the first step, we selected studies with descriptions of AVSS or systems and excluded: (1) basic experiments (in vitro and animal) and basic research (pathology); (2) vaccine management and economics studies, such as policy, health economics, and sociomedical assessment, quality of life; (3) case reports, case series studies, nursing experiences, which primarily described the characteristics of individual patients or small groups; (4) vaccine effectiveness evaluation; (5) studies not in English; (6) studies belonging to other categories; and (7) uncertain studies, requiring full-text reading for clarification in the next step.

During the secondary step, we included only postmarketing association studies, such as cohort studies and case-control studies that were conducted with control groups from the same source population, regardless of whether databases were used. The following publications were excluded: (1) passive surveillance, (2) clinical trials, (3) theoretical or methodological studies for vaccine safety surveillance, (4) studies not classified as association studies, including correlational studies, signaling reports, descriptive studies, or those lacking a comparison group, (5) studies not related to vaccine intrinsic safety, (6) nonoriginal studies, such as reviews, conference abstracts, correspondence, errata, or protocols, (7) studies not in English, or (8) studies without full text. Table 1 presents our inclusion and exclusion criteria.

**Table 1.** Inclusion or exclusion criteria.

Category	Inclusion criteria	Exclusion criteria
Article type	Active surveillance Original studies	Passive surveillance Nonoriginal studies (reviews, abstracts, etc)
Study type	Association studies (cohort and case-control studies)	Basic experiments (in vitro and animal) and basic research (pathology) Vaccine management and economics studies Case reports, case series studies, and nursing experiences Vaccine effectiveness evaluation Clinical trials Theoretical or methodological studies on vaccine safety surveillance Studies not classified as association studies Studies unrelated to vaccine intrinsic safety
Language	English	Non-English studies

For the above steps, 2 trained researchers independently reviewed the title and abstract of each paper, if any disagreement occurred, a senior researcher would participate in the discussion and reach a final agreement.

### Data Analysis

To formulate a standard, reasonable, and validated MDS for AVSS, 3 stages were undertaken to ensure its scientific and practical validity. First, a preliminary framework for the MDS was developed by referencing relevant variables from the Council for International Organizations of Medical Sciences (CIOMS) Guide to Active Vaccine Safety Surveillance and the *COVID-19 Vaccines: Safety Surveillance Manual* [25,26]. This initial framework comprised four dimensions: “Vaccine,” “Outcome,” “Demographic Data,” and “Covariate.” In addition, metadata including data sources, encoding, quality control, and assurance were also taken into consideration. Second, to improve the rationality and feasibility of the drafted framework, experts from diverse fields including immunization, epidemiology, clinical medicine, and databases were consulted to review the framework. A total of 30 eligible studies were randomly selected to refine the framework. Third, a standardized questionnaire for information extraction was developed based on the validated framework. Relevant variables were extracted from the methodology, results, and limitations sections of each study. During this process, any overlooked elements were identified and supplemented through discussion to finalize the framework. Furthermore, basic characteristics of the included studies (authors, publication year, country or region, study design, and sample size) were also collected.

The data extraction and management were completed parallelly by 2 trained researchers using Microsoft Excel 2019, with oversight from a senior researcher responsible for quality control. Subsequently, descriptive statistical analysis was performed using R 4.3.2 (R Core Team), and data visualization was carried out using both R 4.3.2 and OriginPro (Origin Laboratories) 2021.

In the study, the key steps from study design, literature searching, framework formulation, information extraction, and analysis to interpretation have been supervised by external experts to strictly ensure high quality. Meanwhile, as for literature selection and data extraction, every participant was trained and qualified, and the information was double entry independently and parallelly, and any disagreement was ruled out by another senior researcher.

## Results

**Figure 1.**
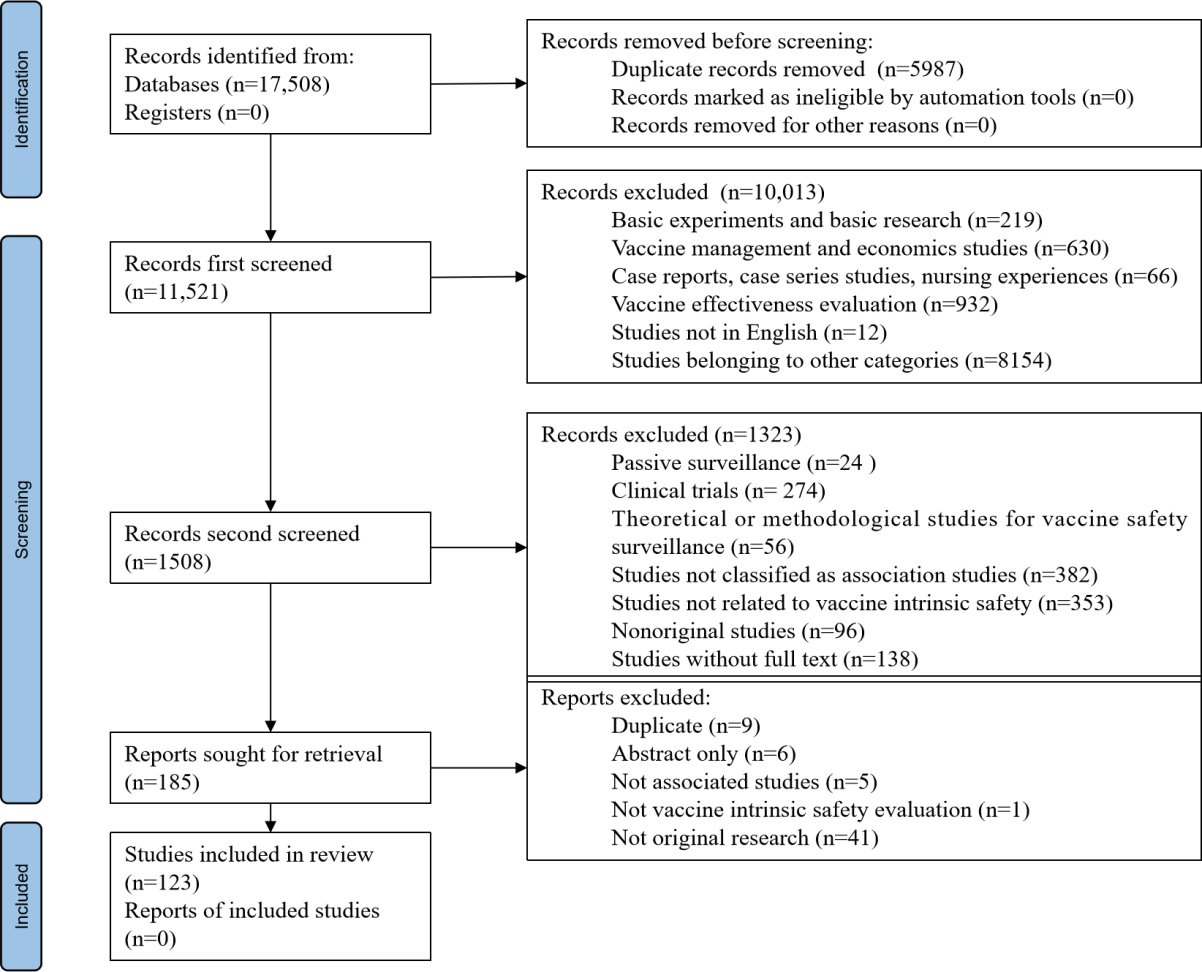
Preferred Reporting Items for Systematic reviews and Meta-Analyses flow diagram.

Initially, 11,512 papers were identified after removing duplicated records. With 2 rounds of literature screening, 185 papers were evaluated in full text. Among them, 9 papers were excluded for duplication, 5 papers were excluded for not meeting the criteria for association studies, 1 paper was excluded due to its failure to evaluate vaccine intrinsic safety, 41 papers were excluded for not being original research, and 6 papers were excluded due to the lack of full-text availability. A total of 123 studies [14,15,17,28-147] were finally included. The inclusion and exclusion process is shown in Figure 1.[Bibr R15]

The general characteristics of eligible studies are shown in Table 2, with more specific details provided in Table S1 in Multimedia Appendix 1. Of the eligible studies, 82.9% (102/123) were cohort studies, while case-control studies accounted for 17.1% (21/123). Regarding vaccines, 39.5% (49/124) investigated the association between the COVID-19 vaccine and adverse events, while 20.2% (25/124) focused on the influenza vaccine. As for the population, 20% (25/125) focused on children or adolescents, 14.4% (18/125) on pregnant women, 10.40% (13/125) on infants, and 4.8% (6/125) on the older people. In addition, 16.0% (20/125) of studies explored vaccine safety in patients with special diseases such as cancer and inflammatory bowel disease.

**Table 2.** General characteristics of selected studies.

Characteristics	Values, n (%)
Country or area	
High-income countries	98 (79.7)
Low- and middle-income countries	16 (13)
Multicountry settings	9 (7.3)
Study design	
Cohort study	102 (82.9)
Case-control study	21 (17.1)
Vaccine^a^	
COVID-19 vaccine	49 (39.5)
Influenza vaccine	25 (20.2)
Tdap^b^	9 (7.3)
HPV^c^	6 (4.8)
Zoster Vaccine	5 (4)
Others	30 (24.2)
Study population^a^	
General population	43 (34.4)
Children and Adolescents	25 (20)
Special patients^d^	20 (16)
Maternal	18 (14.4)
Infants	13 (10.4)
Older people	6 (4.8)

a Some studies involved multiple populations, multivaccines, or multioutcomes.

b Tdap: tetanus, diphtheria, acellular pertussis vaccine .

c HPV: human papillomavirus vaccine.

d Patients with inflammatory bowel disease, cancer, severe influenza, juvenile idiopathic arthritis, asthma, or other diseases.

**Figure 2.**
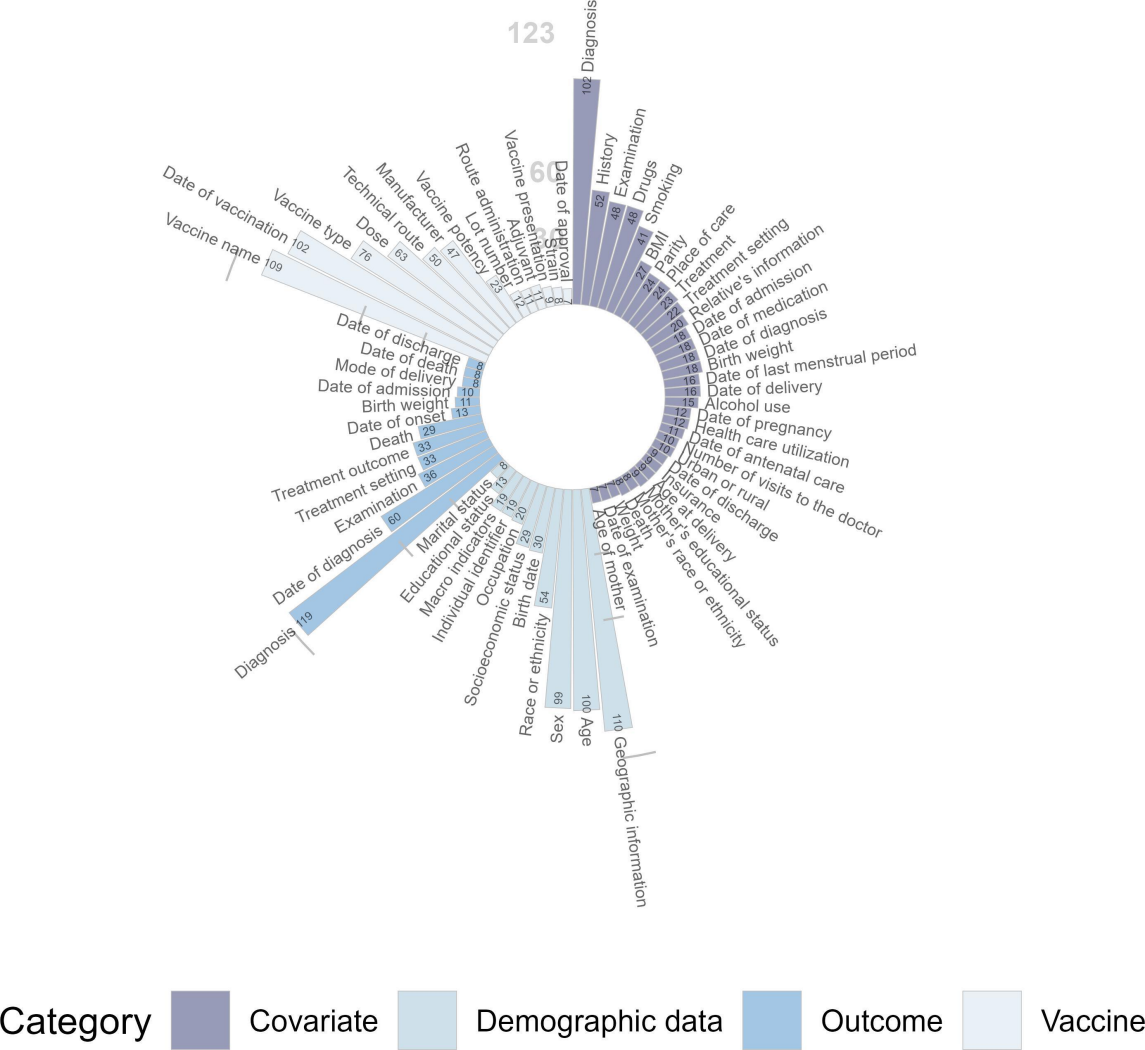
Frequency of Minimum Data Set variables.

Figure 2 illustrates the utilization of the summarized MDS across 123 included studies. Among the categories, “Diagnosis” within the “Outcome” and “Covariate” dimensions appeared most frequently, with 119 occurrences (96.8%) and 102 occurrences (82.9%), respectively. In the “Demographic Data” dimension, “Geographic Information” was the most frequently reported variable, appearing in 110 studies (89.4%). Similarly, “Vaccine Name” was the most commonly used variable in the “Vaccine” dimension, with 109 occurrences (88.6%). Table 3 shows the differences between the CIOMS and WHO guidelines and the application of both in the included studies. The MDS includes almost all variables recommended in the CIOMS and WHO guidelines, along with some additional variables. These extra variables include “Birth Weight,” “Examination,” and “Mode of Delivery” under the “Outcome” category; “Geographic Information” and “Race or Ethnicity” under the “Demographic Data” category; “Technical Route” and “Adjuvant” under the “Vaccine” category; and several additional variables within the “Covariate” category. The details for MDS are shown in Table S2 in Multimedia Appendix 1.

**Table 3.** Comparison among the Council for International Organizations of Medical Sciences guidelines, and the World Health Organization guidelines.

Variables	CIOMS^a^ guidelines	WHO^b^ guidelines	Value, n (%)
Vaccine data			
Place of vaccination	✓	✓	1 (0.8)
Vaccine type	✓	✓^c^	77 (62.6)
Vaccine presentation, single or multiple dose	✓		9 (7.3)
Manufacturer	✓		47 (38.2)
Lot number (of vaccine and any diluents)	✓	✓^c^	12 (9.8)
Date of vaccination (and perhaps time)	✓	✓^c^	102 (82.9)
Vaccine injection site	✓	✓^c^	5 (4.1)
Number of dose	✓	✓^c^	63 (51.2)
Vaccine antigens		✓	7 (5.7)
Concomitant vaccines		✓	5 (4.1)
Route administration		✓	11 (8.9)
Health events or outcomes data			
Place of care	✓	✓	1 (0.8)
Diagnosis(es) or adverse event(s) or outcome	✓	✓^c^	119 (96.8)
Date (and time) of onset of (first) symptom of the event	✓	✓^c^	13 (10.6)
Serious		✓^c^	15 (12.2)
Demographic data			
Age at onset		✓^c^	100 (81.3)
Gender		✓^c^	99 (80.5)
Medical conditions		✓^c^	52 (42.3)
Medication		^c^✓	48 (39)

a CIOMS: Council for International Organizations of Medical Sciences

b WHO: World Health Organization

c The core dataset of the WHO guidelines.

Among the 49 studies focusing on COVID-19 vaccines, a total of 95 variables were identified, of which 54 variables appeared at least 3 times (frequency >5%) and were included in the MDS for COVID-19 vaccines. These variables were distributed as follows: 14 under the “Outcome” category, 10 under “Demographic Data,” 9 under “Vaccine,” and 21 under “Covariate.” In 18 studies targeting the pregnant population, 96 variables were identified, all of which were included in the MDS for this specific population (frequency >5%). These variables were categorized as follows: 20 under “Outcome,” 11 under “Demographic Data,” 11 under “Vaccine,” and 54 under “Covariate.” More details are shown in Tables S3 and S4 in Multimedia Appendix 1.

**Figure 3.**
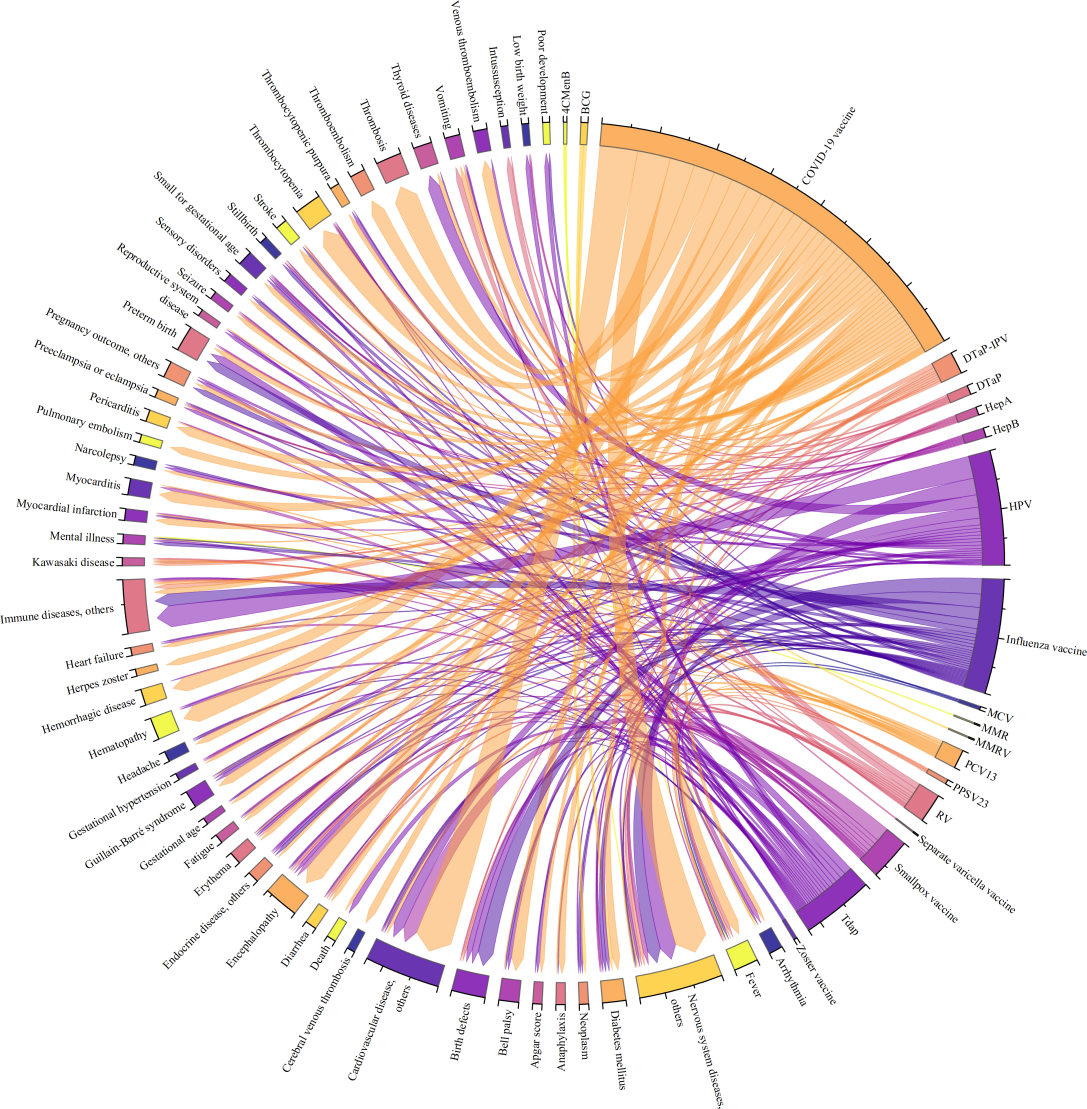
Frequency of "vaccine-outcome" pairs in included studies. 4CMenB: 4-Component group B meningococcal vaccine; BCG: Bacillus Calmette-Guerin vaccine; DTaP-IPV: Diphtheria, tetanus, acellular pertussis, and inactivated poliovirus combination vaccine; DTaP: Diphtheria, tetanus, acellular pertussis vaccine; HepA: Hepatitis A vaccine; HepB: Hepatitis B vaccine; HPV: Human papillomavirus vaccine; MCV: Measles and pertussis-containing vaccine; MMR: Measles, mumps, rubella vaccine; MMRV: Measles, mumps, rubella and varicella vaccine; PCV13: 13-Valent pneumococcal conjugate vaccine; PPSV-23: 23-Valent pneumococcal polysaccharide vaccine; RV: Rotavirus vaccine; Tdap: Diphtheria, tetanus, acellular pertussis vaccine.

Figure 3 plotted the evaluation of the relationship between adverse events and vaccines. We only included diseases that appear more than five times, specifically cardiovascular disease, endocrine diseases, hemorrhagic diseases, nervous system diseases, mental illness, and immune diseases, and we consider the occurrences of outcomes to be counted twice if a publication studies two vaccines. A total of 1239 records specifying health outcomes and vaccine types were identified across 116 studies. The top 5 rare or severe adverse events, each occurring more than 20 times, were encephalopathy (n=30), birth defects (n=29), preterm birth (n=22), thrombocytopenia (n=22), and thrombosis (n=22). Adverse events with fewer than 5 (1%) occurrences were relatively concentrated in 3 groups of diseases: nervous system (n=74), cardiovascular system (n=65), and immune system (n=46). As for COVID-19 vaccines, the top 3 adverse events pairs were thrombosis (n=22), thrombocytopenia (n=19), and encephalopathy (n=17).

A total of 17 studies were completed using the AVSS system, with 11 using the VSD project and the remaining 6 derived from various sources, including the Royal College of General Practitioners (RCGP) Research and Surveillance Centre (RSC), Pharmacoepidemiological Research in Primary Care (Base de datos para la Investigación Farmacoepidemiológica en Atención Primaria; BIFAP), Norway’s emergency preparedness register for COVID-19 (Beredskapregisteret for COVID-19; Beredt C19), Post-Licensure Rapid Immunization Safety Monitoring (PRISM)–Sentinel, the Paediatric Active Enhanced Disease Surveillance network, and the Defense Medical Surveillance System. All these studies explicitly reported the standard codes for outcomes or events. Specifically, VSD used National Drug Codes and *International Classification of Diseases* (*ICD*) codes, PRISM-Sentinel used Current Procedural Terminology, BIFAP and Beredt C19 used *ICD* codes, and RCGP RSC applied 5-byte Read or Clinical Terms Version 3 codes. However, only 8 studies (9.3%) documented their quality control processes. Among these, 3 studies validated diagnoses in the database by assessing the positive predictive value, 2 confirmed data reliability by comparing original records with database entries, 2 used high-quality data that had undergone prior evaluation, and one maintained data quality through regular monitoring.

## Discussion

### Principal Findings

This study is the first to systematically clarify the minimum data requirements for AVSS by conducting a comprehensive review aligned with WHO and CIOMS guidelines, focusing on minimizing data range. The proposed MDS and Metadata framework for AVSS encompasses four key dimensions: “Vaccine,” “Outcome,” “Demographic Data,” and “Covariate.” Across these dimensions, 68 variables were identified: 13 in “Vaccine,” 12 in “Outcome,” 11 in “Demographic Data,” and 32 in “Covariate.” Certain variables were deemed essential for all vaccine safety association studies, including unique and anonymized individual identifiers; vaccine name and vaccination date from the “Vaccine” category; diagnosis and diagnostic date from the “Outcome” category; age and sex from the “Demographic Data” category; and history and drugs from the “Covariate” category. These aspects of data collection, quality control, and assurance must be transparently declared in AVSS protocols; however, regrettably, only a minority of studies addressed them adequately. Therefore, we strongly advocate for the establishment of a globally standardized and widely recognized MDS and Metadata framework. Such a standard would enable countries and regions, particularly LMICs, to rapidly develop AVSS systems or conduct related research. In addition to facilitating data comparability, this effort would significantly strengthen the global vaccine safety ecosystem.

The study design was carefully developed to ensure the reliability and representativeness of the findings. First, the study population covered the whole life course, and the number of studies from various groups, including the general population (43/125, 34.4%), maternal and infants (31/125, 24.8%), children (25/125, 20%), and special patients (20/125, 16%) was relatively balanced. Second, the investigated vaccines spanned a wide spectrum, comprising newly emergency-marketed COVID-19 vaccines, annually administered influenza vaccines, globally disseminated human papillomavirus vaccines, diphtheria, tetanus, acellular pertussis vaccines that often raise concerns, and more than 15 other vaccines in total. Finally, this review spanned over 23 countries and regions, with a notable proportion of 13% (n=16) of included studies from LMICs.

The 49 studies examining the safety of COVID-19 vaccines encompassed diverse populations, including the general population, infants, children and adolescents, pregnant women, and special patients. Despite this breadth, only 54 variables were used, likely reflecting the challenges of limited data availability during the rollout of an emergency vaccine. Previous studies have underscored several challenges in evaluating COVID-19 vaccine safety, including insufficient sample sizes and lower study quality [148-150]. Furthermore, misleading evidence has, at times, hindered efforts to increase vaccine coverage [151]. It is imperative for us to establish a stable AVSS system to provide reliable and sufficient data for the safety studies of emergency vaccines.

The maternal population, which includes 96 variables, reflects a broad spectrum of data, likely due to the inclusion of infant-related variables at this level. Pregnant women are frequently excluded from randomized controlled trials of drug safety because of their unique physiological characteristics [152]. Therefore, the real-world evidence from postmarketing surveillance plays a critical role in evaluating vaccine safety for this population. Furthermore, establishing continuous active surveillance systems facilitates the investigation of the long-term effects of prenatal exposure on offspring.

The standard MDS could provide precise guidance and concise extents of data requirements for the AVSS system. However, no relevant guidelines have yet been released. Although both the CIOMS and WHO guidelines briefly outline the data elements of AVSS, their contents lack consistency and empirical validation in real-world applications [153]. This study is the first to validate these concepts and develop a comprehensive framework. When compared with the common data models of established systems like the VSD and Sentinel, the MDS shares several core strengths. Like these systems, the MDS encompasses the entire lifecycle population, covers a wide array of vaccines, and accounts for a broad spectrum of events. Furthermore, the MDS in our study demonstrated the other two advantages. First, it focuses exclusively on vaccine safety surveillance, minimizing unnecessary data collection efforts and addressing the resource constraints often faced in such endeavors. Second, the MDS incorporates a wider range of data sources, including registration and survey data, which better reflect the diversity of real-world settings and enhance the representativeness of AVSS systems.

A well-defined MDS is crucial for ensuring data quality, comparability, and efficient monitoring in public health surveillance. Within an AVSS system, an effective MDS helps identify key indicators, enhances data accessibility, and strengthens risk assessment [22]. By standardizing data collection, it reduces fragmentation, ensures dataset consistency, and enables timely, automated signal detection, allowing policymakers to respond swiftly to emerging safety concerns. In resource-constrained regions, prioritizing key variables such as vaccination date, vaccine name, outcome diagnosis, and diagnosis date, among the most frequently reported in our study, ensures feasibility while maintaining surveillance effectiveness. From a public health perspective, our study aims to establish a robust AVSS that balances data accessibility and efficiency. By integrating elements from established frameworks such as CIOMS and WHO guidelines, we validate their applicability through a comprehensive literature review. Our findings offer a foundational reference for resource-limited regions, with future efforts focusing on refining MDS definitions through expert consultation and real-world application.

Metadata, which reflects data quality and applicability, is essential for ensuring that real-world data can generate real-world evidence. Unfortunately, only 14.6% of the 103 studies on databases provided Metadata information, indicating that most studies overlooked the importance of data quality control and assurance. This raises concerns about the overall quality of evidence in AVSS from previous studies. It’s imperative to increase awareness and provide clear guidelines in the future. Certainly, the MDS and Metadata are essential components of the information infrastructure for scientific research on vaccine safety vigilance. Prioritizing their importance ensures the establishment of high-quality evidence, enhances evidence transparency, and facilitates evidence translation, particularly in LMICs [154].

There were several limitations in our study. First, our review focused exclusively on association studies in vaccine safety from the past 5 years, which may only represent the latest progress in this field but not its full picture. Second, the variables mentioned in previous studies may not fully correspond to those actually used, potentially introducing some bias in the frequency description. Third, non-English studies were excluded from our analysis. However, given that AVSS studies mostly originate from high-income countries, the impact of this publication bias is likely minimal and can be disregarded. Fourth, our analysis was limited to comparative observational studies (eg, cohort studies and case-control studies) with explicit comparison groups, excluding self-controlled designs (eg, self-controlled case series, and case-crossover studies). While this exclusion represents a limitation, Self-Controlled Case Series (SCCS) studies typically require a smaller set of predefined variables, and the key variables used in SCCS designs are already encompassed within cohort and case-control studies. Therefore, although excluding SCCS studies may slightly reduce the frequency counts of certain variables, it does not affect the overall data scope of the identified MDS. Fifth, this study included variables with a frequency of ≥5% as part of the MDS. While variables with <5% frequency, although less commonly reported, may still hold significant importance, such as “place of vaccination” for detecting clustering of adverse events. For transparency and future reference, a complete list of variables identified in this study is provided in Table S5 in Multimedia Appendix 1.

### Conclusion

In conclusion, following WHO and CIOMS guidance and adhering to the Preferred Reporting Items for Systematic Reviews and Meta-Analyses (PRISMA) guidelines (Checklist 1), this study presents an initial framework of MDS and Metadata for AVSS. This MDS can provide precise guidance and concise requirements for the data scope of the AVSS system and studies. This framework is particularly valuable for resource-limited regions, providing a foundation for implementing AVSS systems effectively and efficiently. Therefore, it is crucial to establish a standardized MDS globally based on these findings to enhance the global vaccine safety ecosystem.

## Supplementary material

10.2196/63161 Multimedia Appendix 1 Basic information and extracted data elements of included studies.

10.2196/63161 Checklist 1 PRISMA (Preferred Reporting Items for Systematic Reviews and Meta-Analyses) 2020 checklist.

## Abbreviations

AVSS: active vaccine safety surveillance
Beredt C19: Beredskapregisteret for COVID-19
BIFAP: Base de datos para la Investigación Farmacoepidemiológica en Atención Primaria
CIOMS: Council for International Organizations of Medical Sciences guidelines
FDA: Food and Drug Administration
IA2030: Immunization Agenda 2030
*ICD*: *International Classification of Diseases*
LMIC: low-income and middle-income country
MDS: Minimum Data Set
PRISM: Post-Licensure Rapid Immunization Safety Monitoring
PRISMA: Preferred Reporting Items for Systematic Reviews and Meta-Analyses
RCGP: Royal College of General Practitioners
RSC: Research and Surveillance Centre
SCCS: Self-Controlled Case Series
VSD: Vaccine Safety Datalink
WHO: World Health Organization
