# Supplementary material for: Minimum Data Set and Metadata for Active Vaccine Safety Surveillance: Systematic Review
Source: JMIR Public Health Surveill. 2025 Jun 17;11:e63161. doi: 10.2196/63161 (PMC12187031; doi:10.2196/63161)
Supplement: Multimedia Appendix 1 [file publichealth-v11-e63161-s001.pdf]

# Contents

**Search Strategy** ........ 2

**Supplementary Table 1: Characteristics of Selected Studies** ........ 4

**Supplementary Table 2: Minimum Data Sets** ........ 15

**Supplementary Table 3: Minimum Data Sets for COVID-19 vaccines** ........ 17

**Supplementary Table 4: Minimum Data Sets for Pregnant Women** ........ 19

**Supplementary Table 5: All variables collected** ........ 22

# Search Strategy

**1.PubMed**

**#1:** vaccination[Title/Abstract] OR Vaccines[Title/Abstract] OR immunization programs[Title/Abstract] OR immunization[Title/Abstract] OR vaccin*[Title/Abstract] OR immuniz*[Title/Abstract]

**#2:** vaccination[Mesh] OR Vaccines[Mesh] OR "immunization programs"[Mesh] OR immunization[Mesh]

**#3:** safety[Title/Abstract] OR adverse effects[Title/Abstract] OR Drug-Related Side Effects and Adverse Reactions[Title/Abstract] OR Injection Site Reaction[Title/Abstract] OR Long Term Adverse Effects[Title/Abstract] OR Adverse Drug Reaction[Title/Abstract] OR Risk Management[Title/Abstract] OR Safety Management[Title/Abstract] OR Risk[Title/Abstract] OR ADRs[Title/Abstract] OR safe[Title/Abstract] OR pharmacovigilance[Title/Abstract]

**#4**: safety[Mesh] OR "adverse effects"[Mesh] OR "Drug-Related Side Effects and Adverse Reactions"[Mesh] OR "Injection Site Reaction"[Mesh] OR "Long Term Adverse Effects"[Mesh] OR "Adverse Drug Reaction Reporting Systems"[Mesh] OR "Risk Management"[Mesh] OR "Safety Management"[Mesh] OR Risk[Mesh] OR pharmacovigilance[Mesh]

**#5**: "rare event*"[Title/Abstract] OR "rare diseas*"[Title/Abstract] OR "adverse outcome*"[Title/Abstract] OR "adverse reaction*"[Title/Abstract] OR "adverse event*"[Title/Abstract] OR "detrimental effect*"[Title/Abstract]

**#6**: Cohort[Title/Abstract] OR Birth Cohort[Title/Abstract] OR Case-Control[Title/Abstract]

**#7**: "Cohort Studies"[Mesh] OR "Birth Cohort"[Mesh] OR "Case-Control Studies"[Mesh]

**#8**: "Minimum data"[Title/Abstract] OR "Minimum dataset*"[Title/Abstract] OR "Minimum set*" [Title/Abstract] OR "basic data"[Title/Abstract] OR "minimal data"[Title/Abstract] OR "minimal dataset"[Title/Abstract] OR "minimal set*"[Title/Abstract]

**#9**: #1 OR #2

**#10**: #3 OR #4 OR #5

**#11**: #6 OR #7 OR #8

**#12**: #9 AND #10 AND #11

**2. Embase**

**#1:** vaccination OR Vaccines OR ‘immunization programs’ OR immunization OR vaccin* OR immuniz* OR vaccine/exp OR immunization/exp

**#2:** safety OR ‘adverse effects’ OR ‘Drug-Related Side Effects and Adverse Reactions’ OR ‘side effect’/exp OR ‘Injection Site Reaction’ OR ‘Long Term Adverse Effects’ OR ‘Adverse Drug Reaction’ OR ‘Risk Management’ OR ‘Safety Management’ OR Risk OR ADRs OR safe OR ‘rare event*’ OR ‘rare diseas*’ OR ‘adverse outcome*’ OR ‘adverse reaction*’ OR ‘adverse event*’ OR ‘detrimental effect*’ OR ‘adverse event’/exp OR ‘pharmacovigilance’

**#3**: ‘Cohort analysis’ OR ‘Birth Cohort’ OR ‘Case-Control’ OR ‘case control study’/exp OR ‘Minimum data’ OR ‘Minimum dataset*’ OR ‘Minimum set*’ OR ‘basic data’ OR ‘minimal data’ OR ‘minimal dataset’ OR ‘minimal set*’

**#4**: #1 AND #2 AND #3

**3. Web of science**

**#1:** vaccination OR Vaccines OR immunization programs OR immunization OR vaccin* OR immuniz* (Title) OR vaccination OR Vaccines OR immunization programs OR immunization OR vaccin* OR

immuniz* (Abstract)

**#2:** pharmacovigilance OR safety OR adverse effects OR Drug-Related Side Effects and Adverse Reactions OR Injection Site Reaction OR Long Term Adverse Effects OR Adverse Drug Reaction OR Risk Management OR Safety Management OR Risk OR ADRs OR safe OR "rare event*" OR "rare diseas*" OR "adverse outcome*" OR "adverse reaction*" OR "adverse event*" OR "detrimental effect*" (Title) OR pharmacovigilance OR safety OR adverse effects OR Drug-Related Side Effects and Adverse Reactions OR Injection Site Reaction OR Long Term Adverse Effects OR Adverse Drug Reaction OR Risk Management OR Safety Management OR Risk OR ADRs OR safe OR "rare event*" OR "rare diseas*" OR "adverse outcome*" OR "adverse reaction*" OR "adverse event*" OR "detrimental effect*" (Abstract)

**#3:** Cohort OR Birth Cohort OR Case-Control OR "Minimum data" OR "Minimum dataset" OR "Minimum set*" OR "basic data" OR "minimal data" OR "minimal dataset" OR "minimal dataset" OR "minimal set*" (Title) OR Cohort OR Birth Cohort OR Case-Control OR "Minimum data" OR "Minimum dataset" OR "Minimum set*" OR "basic data" OR "minimal data" OR "minimal dataset" OR "minimal dataset" OR "minimal set*" (Abstract)

**#4**: #3 AND #2 AND #1

**Supplementary Table 1: Characteristics of Selected Studies**

| No. | Author, Year | Title | Area | Primary Design | Secondary Design | Sample Size |
|---|---|---|---|---|---|---|
| 1 | Cross et al, 2020 | Adverse events of interest vary by influenza vaccine type and brand: Sentinel network study of eight seasons (2010–2018) | The United Kingdom | Cohort study | SCCS study | 848375 |
| 2 | Alfayadh et al, 2020 | Vaccinations Do Not Increase Arthritis Flares in Juvenile Idiopathic Arthritis: A Study of the Relationship between Routine Childhood Vaccinations on the Australian Immunisation Schedule and Arthritis Activity in Children with Juvenile Idiopathic Arthritis | Australia | Cohort study | SCCS study | 138 |
| 3 | Rolfes et al, 2019 | Measurement of birth outcomes in analyses of the impact of maternal influenza vaccination | Lao People's Democratic Republic | Cohort study | - | 4854 |
| 4 | Larsson et al, 2017 | Pandemrix® vaccination is not associated with increased risk of islet autoimmunity or type 1 diabetes in the TEDDY study children | Multi-country | Cohort study | - | 3401 |
| 5 | Corsenac et al, 2022 | Early life Bacillus Calmette-Guerin vaccination and incidence of type 1, type 2, and latent autoimmune diabetes in adulthood | Canada | Cohort study | - | 396118 |
| 6 | Caspard et al, 2018 | Evaluation of the safety of live attenuated influenza vaccine (LAIV) in children and adolescents with asthma and high-risk conditions: a population based prospective cohort study conducted in England with the Clinical Practice Research Datalink | The United Kingdom | Cohort study | - | 11463 |
| 7 | Yamamoto-Hanada et al, 2020 | Cumulative inactivated vaccine exposure and allergy development among children: a birth cohort from Japan | Japan | Cohort study | - | 56277 |
| 8 | Salmon et al, 2019 | Association between Bacillus Calmette–Guerin vaccination and lymphoma: a population-based birth cohort study | Canada | Cohort study | - | 388040 |
| 9 | Øland et al, 2021 | Reduced Mortality After Oral Polio Vaccination and Increased Mortality After Diphtheria-tetanus-pertussis Vaccination in Children in a Low-income Setting | Guinea-Bissau | Cohort study | - | 1184 |
| 10 | Gögenur et al, 2021 | Association of influenza vaccine and risk of recurrence in patients undergoing curative surgery for colorectal cancer | Denmark | Cohort study | - | 9869 |

| No. | Author, Year | Title | Area | Primary Design | Secondary Design | Sample Size |
|---|---|---|---|---|---|---|
| 11 | Christiansen et al, 2019 | Influenza vaccination and 1-year risk of myocardial infarction, stroke, heart failure, pneumonia, and mortality among intensive care unit survivors aged 65 years or older: a nationwide population-based cohort study | Denmark | Cohort study | - | 89818 |
| 12 | Orta et al, 2020 | A prospective study of influenza vaccination and time to pregnancy | Multi-country | Cohort study | - | 10791 |
| 13 | Hall et al, 2020 | Safety of tetanus, diphtheria, and acellular pertussis vaccination among pregnant active duty U.S. military women | the United States | Cohort study | - | 145883 |
| 14 | Proanos et al, 2021 | Prior influenza vaccine is not a risk factor for bacterial coinfection in patients admitted to the ICU due to severe influenza | Spain | Cohort study | - | 4175 |
| 15 | Knowlton et al, 2022 | A Small but Significantly Greater Incidence of Inflammatory Heart Disease Identified After Vaccination for Severe Acute Respiratory Syndrome Coronavirus 2 | the United States | Cohort study | SCCS study | 67 |
| 16 | Horvat et al, 2022 | Frequency and outcomes of MRI-detected axillary adenopathy following COVID-19 vaccination | the United States | Cohort study | - | 357 |
| 17 | Peretz-Machluf et al, 2022 | Obstetric and Neonatal Outcomes following COVID-19 Vaccination in Pregnancy | Israel | Cohort study | - | 3700 |
| 18 | Whiteley et al, 2022 | Association of COVID-19 vaccines ChAdOx1 and BNT162b2 with major venous, arterial, or thrombocytopenic events: A population-based cohort study of 46 million adults in England | The United Kingdom | Cohort study | - | 46162942 |
| 19 | Toepfner et al, 2022 | Comparative Safety of the BNT162b2 Messenger RNA COVID-19 Vaccine vs Other Approved Vaccines in Children Younger Than 5 Years | Germany | Cohort study | - | 7806 |
| 20 | Chou et al, 2022 | Comparisons of the risk of myopericarditis between COVID-19 patients and individuals receiving COVID-19 vaccines: a population-based study | Hong Kong, China | Cohort study | - | 11441 |
| 21 | Becerra-Culqui et al, 2022 | Prenatal Influenza Vaccination or Influenza Infection and Autism Spectrum Disorder in Offspring | the United States | Cohort study | - | 84739 mother-child pairs |

| No. | Author, Year | Title | Area | Primary Design | Secondary Design | Sample Size |
|---|---|---|---|---|---|---|
| 22 | Petousis-Harris et al, 2019 | A Retrospective Cohort Study of Safety Outcomes in New Zealand Infants Exposed to Tdap Vaccine in Utero | New Zealand | Cohort study | - | 69389 |
| 23 | Wesselink et al, 2022 | A Prospective Cohort Study of COVID-19 Vaccination, SARS-CoV-2 Infection, and Fertility | Multi-country | Cohort study | - | 2126 couples |
| 24 | Weaver et al, 2022 | Impact of SARS-CoV-2 Vaccination on Inflammatory Bowel Disease Activity and Development of Vaccine-Related Adverse Events: Results From PREVENT - COVID | the United States | Cohort study | - | 3316 |
| 25 | Tseng et al, 2018 | Pneumococcal Conjugate Vaccine Safety in Elderly Adults | the United States | Cohort study | - | 545727 Doses* |
| 26 | Agger et al, 2021 | Increased Incidence of Giant Cell Arteritis After Introduction of a Live Varicella Zoster Virus Vaccine | the United States | Cohort study | - | 71008 |
| 27 | Tseng et al, 2022 | Safety of tetanus, diphtheria, acellular pertussis (Tdap) vaccination during pregnancy | the United States | Cohort study | - | 33212 |
| 28 | Andrews et al, 2022 | Risk of venous thrombotic events and thrombocytopenia in sequential time periods after ChAdOx1 and BNT162b2 COVID-19 vaccines: A national cohort study in England | The United Kingdom | Cohort study | - | 27378384 |
| 29 | Corrao et al, 2022 | Balancing Benefits and Harms of COVID-19 Vaccines: Lessons from the Ongoing Mass Vaccination Campaign in Lombardy, Italy | Italy | Cohort study | - | 2381883 |
| 30 | Hoffmann et al, 2022 | Risk factors for granulomas in children following immunization with aluminium-adsorbed vaccines: A Danish population-based cohort study | Denmark | Cohort study | - | 553932 |
| 31 | Baldolli et al, 2022 | Reactogenicity among health care workers following a BNT162b2 or mRNA-1273 second dose after priming with a ChAdOx1 nCOV-19 vaccine | France | Cohort study | - | 356 |
| 32 | Zerbo et al, 2022 | Safety of measles and pertussis-containing vaccines in children with autism spectrum disorders | the United States | SCCS study | Cohort study | 1650041 |

| No. | Author, Year | Title | Area | Primary Design | Secondary Design | Sample Size |
|---|---|---|---|---|---|---|
| 33 | Taquet et al, 2021 | Cerebral venous thrombosis and portal vein thrombosis: A retrospective cohort study of 537,913 COVID-19 cases | the United States | Cohort study | - | 537913 |
| 34 | Foo et al, 2022 | Maternal influenza vaccination and child mortality: Longitudinal, population-based linked cohort study | Australia | Cohort study | - | 191247 mother-child pairs |
| 35 | Ou et al, 2021 | Safety and Reactogenicity of 2 Doses of SARS-CoV-2 Vaccination in Solid Organ Transplant Recipients | the United States | Cohort study | - | 741 |
| 36 | Martín-Merino et al, 2021 | Papillomavirus vaccination and Guillain-Barre Syndrome among girls: A cohort study in Spain | Spain | Cohort study | - | 388849 |
| 37 | Pottegård et al, 2021 | Arterial events, venous thromboembolism, thrombocytopenia, and bleeding after vaccination with Oxford-AstraZeneca ChAdOx1-S in Denmark and Norway: population based cohort study | Multi-country | Cohort study | - | 281264 |
| 38 | Blakeway et al, 2022 | COVID-19 vaccination during pregnancy: coverage and safety | The United Kingdom | Cohort study | - | 1328 |
| 39 | Dick et al, 2022 | Safety of third SARS-CoV-2 vaccine (booster dose) during pregnancy | Israel | Cohort study | - | 6507 |
| 40 | Bartels et al, 2021 | Local and systemic reactogenicity of COVID-19 vaccine BNT162b2 in patients with systemic lupus erythematosus and rheumatoid arthritis | Denmark | Cohort study | - | 282 |
| 41 | Karlstad et al, 2022 | SARS-CoV-2 Vaccination and Myocarditis in a Nordic Cohort Study of 23 Million Residents | Multi-country | Cohort study | - | 23122522 |
| 42 | Speake et al, 2021 | Risk of adverse maternal and foetal outcomes associated with inactivated influenza vaccination in first trimester of pregnancy | Australia | Cohort study | - | 70838 |
| 43 | Hviid et al, 2019 | Measles, Mumps, Rubella Vaccination and Autism: A Nationwide Cohort Study | Denmark | Cohort study | - | 651467 |

| No. | Author, Year | Title | Area | Primary Design | Secondary Design | Sample Size |
|---|---|---|---|---|---|---|
| 44 | Foo et al, 2022 | Prenatal influenza vaccination and allergic and autoimmune diseases in childhood: A longitudinal, population-based linked cohort study | Australia | Cohort study | - | 124760 |
| 45 | Kent et al, 2019 | Safety of meningococcal group B vaccination in hospitalised premature infants | The United Kingdom | Cohort study | - | 201 |
| 46 | Groom et al, 2018 | Uptake and safety of Hepatitis B vaccination during pregnancy: A Vaccine Safety Datalink study | the United States | Cohort study | - | 653831 |
| 47 | Lee et al, 2019 | Post-marketing surveillance to assess the safety and tolerability of a combined diphtheria, tetanus, acellular pertussis and inactivated poliovirus vaccine (DTaP-IPV) in Korean children | Korea | Cohort study | - | 639 |
| 48 | Tu et al, 2022 | Incidence of Cerebral Venous Thrombosis Following SARS-CoV-2 Infection vs mRNA SARS-CoV-2 Vaccination in Singapore | Singapore | Cohort study | - | 3006662 |
| 49 | Dick et al, 2022 | Safety of SARS-CoV-2 vaccination during pregnancy-obstetric outcomes from a large cohort study | Israel | Cohort study | - | 5618 |
| 50 | Becerra-Culqui et al, 2018 | Prenatal Tetanus, Diphtheria, Acellular Pertussis Vaccination and Autism Spectrum Disorder | the United States | Cohort study | - | 81993 |
| 51 | Baker et al, 2019 | Kawasaki disease and 13-valent pneumococcal conjugate vaccination among young children: A self-controlled risk interval and cohort study with null results | the United States | SCCS study | Cohort study | 6177795 Doses* |
| 52 | Laverty et al, 2021 | Health Outcomes in Young Children Following Pertussis Vaccination During Pregnancy | Canada | Cohort study | - | 625643 |
| 53 | Pawlowski et al, 2021 | Cerebral Venous Sinus Thrombosis is not Significantly Linked to COVID-19 Vaccines or Non-COVID Vaccines in a Large Multi-State Health System | the United States | Cohort study | - | 132913 Doses* |
| 54 | Shasha et al, 2021 | Real-world safety data for the Pfizer BNT162b2 SARS-CoV-2 vaccine: historical cohort study | Israel | Cohort study | - | 728384 |
| 55 | Rottenstreich et al, 2021 | Covid-19 vaccination during the third trimester of pregnancy: rate of vaccination and maternal and neonatal outcomes, a multicentre retrospective cohort study | Israel | Cohort study | - | 1775 |

| No. | Author, Year | Title | Area | Primary Design | Secondary Design | Sample Size |
|---|---|---|---|---|---|---|
| 56 | Hviid et al, 2018 | Human papillomavirus vaccination of adult women and risk of autoimmune and neurological diseases | Multi-country | Cohort study | SCCS study | 3126790 |
| 57 | Bardenheier et al, 2021 | Adverse events following mRNA SARS-CoV-2 vaccination among U.S. nursing home residents | the United States | Cohort study | - | 21222 |
| 58 | Scherrer et al, 2022 | Impact of herpes zoster vaccination on incident dementia: A retrospective study in two patient cohorts | the United States | Cohort study | - | 308806 |
| 59 | Arora et al, 2020 | A Prospective Cohort Study on the Safety of Infant Pentavalent (DTwP-HBV-Hib) and Oral Polio Vaccines in Two South Indian Districts | India | Cohort study | - | 30688 |
| 60 | Bruxvoort et al, 2022 | Association Between 2-Dose vs 3-Dose Hepatitis B Vaccine and Acute Myocardial Infarction | the United States | Cohort study | - | 69625 |
| 61 | Mohammed et al, 2021 | Safety of maternal pertussis vaccination on pregnancy and birth outcomes: A prospective cohort study | Australia | Cohort study | - | 1272 |
| 62 | Ludvigsson et al, 2020 | Maternal Influenza A(H1N1) Immunization During Pregnancy and Risk for Autism Spectrum Disorder in Offspring: A Cohort Study | Sweden | Cohort study | - | children:69019 mothers:69002 |
| 63 | Xiong et al, 2022 | Safety of Inactivated and mRNA COVID-19 Vaccination Among Patients Treated for Hypothyroidism: A Population-Based Cohort Study | Hong Kong, China | Cohort study | - | 47086 |
| 64 | Scherrer et al, 2021 | Lower Risk for Dementia Following Adult Tetanus, Diphtheria, and Pertussis (Tdap) Vaccination | the United States | Cohort study | - | 296999 |
| 65 | MacDonald et al, 2018 | Is varicella vaccination associated with pediatric arterial ischemic stroke? A population-based cohort study | Canada | Cohort study | - | 368992 |
| 66 | Ohfuji et al, 2020 | Safety of influenza vaccination on adverse birth outcomes among pregnant women: A prospective cohort study in Japan | Japan | Cohort study | - | 10330 |
| 67 | Wijn et al, 2018 | Influenza vaccination in patients with lung cancer receiving anti-programmed death receptor 1 immunotherapy does not induce immune-related adverse events | Netherlands | Cohort study | - | 127 |

| No. | Author, Year | Title | Area | Primary Design | Secondary Design | Sample Size |
|---|---|---|---|---|---|---|
| 68 | Walsh et al, 2019 | Health outcomes of young children born to mothers who received 2009 pandemic H1N1 influenza vaccination during pregnancy: retrospective cohort study | Canada | Cohort study | - | 104249 |
| 69 | Andersson et al, 2022 | Safety of heterologous primary and booster schedules with ChAdOx1-S and BNT162b2 or mRNA-1273 vaccines: nationwide cohort study | Denmark | Cohort study | - | 5152620 |
| 70 | Barda et al, 2021 | Safety of the BNT162b2 mRNA Covid-19 Vaccine in a Nationwide Setting | Israel | Cohort study | - | 1769656 |
| 71 | Sheel et al, 2022 | Severity of Rotavirus-Vaccine-Associated Intussusception: Prospective Hospital-Based Surveillance, Australia, 2007-2018 | Australia | Cohort study | - | 323 |
| 72 | Hanson et al, 2022 | Incidence of Guillain-Barré Syndrome After COVID-19 Vaccination in the Vaccine Safety Datalink | the United States | Cohort study | - | 7894989 |
| 73 | Decker et al, 2021 | Enhanced safety surveillance study of ACAM2000 smallpox vaccine among US military service members | the United States | Cohort study | - | 897227 |
| 74 | Cunha et al, 2020 | Thimerosal-containing vaccines and deficit in child development: Population-based study in southern Brazil | Brazil | Cohort study | - | Mothers:535<br>Children:535 |
| 75 | Rider et al, 2022 | Baseline factors associated with self-reported disease flares following COVID-19 vaccination among adults with systemic rheumatic disease: results from the COVID-19 global rheumatology alliance vaccine survey | Multi-country | Cohort study | - | 5619 |
| 76 | Layton et al, 2018 | Rotavirus Vaccination and Short-term Risk of Adverse Events in US Infants | the United States | Cohort study | - | 1031431 |
| 77 | Sarna et al, 2022 | The risk of major structural birth defects associated with seasonal influenza vaccination during pregnancy: A population-based cohort study | Australia | Cohort study | - | 124815 |
| 78 | Hertel et al, 2022 | Real-world evidence from over one million COVID-19 vaccinations is consistent with reactivation of the varicella-zoster virus | Multi-country | Cohort study | - | 2190172 |
| 79 | Bukhbinder et al, 2022 | Risk of Alzheimer’s Disease Following Influenza Vaccination: A Claims-Based Cohort Study Using Propensity Score Matching | the United States | Cohort study | - | 2356479 |

| No. | Author, Year | Title | Area | Primary Design | Secondary Design | Sample Size |
|---|---|---|---|---|---|---|
| 80 | van Dongen et al, 2021 | Rotavirus Vaccine Safety and Effectiveness in Infants With High-Risk Medical Conditions | Netherlands | Cohort study | - | 1482 |
| 81 | Peppa et al, 2020 | Seasonal Influenza Vaccination During Pregnancy and the Risk of Major Congenital Malformations in Live-born Infants: A 2010–2016 Historical Cohort Study | The United Kingdom | Cohort study | - | 78150 |
| 82 | Goldshtein et al, 2022 | Association of BNT162b2 COVID-19 Vaccination During Pregnancy With Neonatal and Early Infant Outcomes | Israel | Cohort study | - | 24288 |
| 83 | Kharbanda et al, 2021 | Association of Inadvertent 9-Valent Human Papillomavirus Vaccine in Pregnancy With Spontaneous Abortion and Adverse Birth Outcomes | the United States | Cohort study | - | 1493 |
| 84 | Groom et al, 2019 | Uptake and safety of hepatitis A vaccination during pregnancy: A Vaccine Safety Datalink study | the United States | Cohort study | - | 653826 |
| 85 | Cuschieri et al, 2021 | Adverse reactions to Pfizer- BioNTech vaccination of healthcare workers at Malta's state hospital | Malta | Cohort study | - | 1480 |
| 86 | Yoon et al, 2021 | Association between human papillomavirus vaccination and serious adverse events in South Korean adolescent girls: nationwide cohort study | Korea | Cohort study | SCCS study | 441399 |
| 87 | Klein et al, 2021 | Surveillance for Adverse Events After COVID-19 mRNA Vaccination | the United States | Cohort study | - | 10162227 Doses* |
| 88 | Goud et al, 2021 | Risk of Guillain-Barré Syndrome Following Recombinant Zoster Vaccine in Medicare Beneficiaries | the United States | Cohort study | SCCS study | 2666496 |
| 89 | Lai et al, 2022 | Post-Covid-19-vaccination adverse events and healthcare utilization among individuals with or without previous SARS-CoV-2 infection | Hong Kong, China | Cohort study | - | 1141505 |
| 90 | Kang et al, 2022 | Safety of two-dose COVID-19 vaccination (BNT162b2 and CoronaVac) in adults with cancer: a territory-wide cohort study | Hong Kong, China | Cohort study | - | 50924 |
| 91 | Frisch et al, 2018 | Quadrivalent human papillomavirus vaccination in boys and risk of autoimmune diseases, neurological diseases and venous thromboembolism | Denmark | Cohort study | - | 568410 |

| No. | Author, Year | Title | Area | Primary Design | Secondary Design | Sample Size |
|---|---|---|---|---|---|---|
| 92 | Hertel et al, 2022 | Onset of Oral Lichenoid Lesions and Oral Lichen Planus Following COVID-19 Vaccination: A Retrospective Analysis of about 300,000 Vaccinated Patients | the United States | Cohort study | - | 435726 |
| 93 | Hviid et al, 2021 | Association Between Human Papillomavirus Vaccination and Primary Ovarian Insufficiency in a Nationwide Cohort | Denmark | Cohort study | - | 996300 |
| 94 | Wong et al, 2022 | Adverse events of special interest and mortality following vaccination with mRNA (BNT162b2) and inactivated (CoronaVac) SARS-CoV-2 vaccines in Hong Kong: A retrospective study | Hong Kong, China | Cohort study | - | 2264679 |
| 95 | Houghton et al, 2022 | Risk of venous thromboembolism after COVID- 19 vaccination | the United States | Cohort study | - | 792010 |
| 96 | Faix et al, 2022 | Prospective safety surveillance study of ACAM2000 smallpox vaccine in deploying military personnel | the United States | Cohort study | - | 14667 |
| 97 | Hviid et al, 2022 | Association of AZD1222 and BNT162b2 COVID-19 Vaccination With Thromboembolic and Thrombocytopenic Events in Frontline Personnel | Denmark | Cohort study | - | 355209 |
| 98 | Lai et al, 2022 | Adverse events of special interest following the use of BNT162b2 in adolescents: a population-based retrospective cohort study | Hong Kong, China | Cohort study | - | 512,848 |
| 99 | Becerra-Culqui et al, 2020 | The Association of Prenatal Tetanus, Diphtheria, and Acellular Pertussis (Tdap) Vaccination With Attention-Deficit/Hyperactivity Disorder | the United States | Cohort study | - | 85607 |
| 100 | Simpson et al, 2021 | First-dose ChAdOx1 and BNT162b2 COVID-19 vaccines and thrombocytopenic, thromboembolic and hemorrhagic events in Scotland | The United Kingdom | Cohort study | SCCS study | 2.53 million |
| 101 | Khan et al, 2022 | Safety of Recombinant Zoster Vaccine in Patients with Inflammatory Bowel Disease | the United States | Cohort study | - | 3354 |
| 102 | Failing et al, 2020 | Safety of Influenza Vaccine in Patients With Cancer Receiving Pembrolizumab | the United States | Cohort study | - | 162 |

| No. | Author, Year | Title | Area | Primary Design | Secondary Design | Sample Size |
|---|---|---|---|---|---|---|
| 103 | Kiely et al, 2018 | Investigation of an increase in large local reactions following vaccine schedule change to include DTaP-HB-IPV-Hib (Infanrix-hexa®) and MMRV (ProQuad®) at 18months of age | Canada | Case-control study | - | 590 |
| 104 | Lin et al, 2022 | The Association between Influenza Vaccination and Stroke Risk in Patients with Hypertension: A Nationwide Population-Based Study | Taiwan, China | Case-control study | - | 59251 |
| 105 | Weibel et al, 2018 | Narcolepsy and adjuvanted pandemic influenza A (H1N1) 2009 vaccines – Multi-country assessment | Multi-country | Case-control study | - | 3875 |
| 106 | Yokomichi et al, 2020 | Immune thrombocytopenic purpura risk by live, inactivated and simultaneous vaccinations among Japanese adults, children and infants: a matched case–control study | Japan | Case-control study | - | 175 |
| 107 | Nayak et al, 2019 | Prevalence of Intussusception after Rotavirus Vaccination: A Hospital Based Study from Odisha, India | India | Case-control study | - | 25 |
| 108 | Wan et al, 2022 | Messenger RNA Coronavirus Disease 2019 (COVID-19) Vaccination With BNT162b2 Increased Risk of Bell's Palsy: A Nested Case-Control and Self-Controlled Case Series Study | Hong Kong, China | Case-control study | SCCS study | 5462 |
| 109 | Donahue et al, 2019 | Inactivated influenza vaccine and spontaneous abortion in the Vaccine Safety Datalink in 2012–13, 2013–14, and 2014–15 | the United States | Case-control study | - | 2472 |
| 110 | Oberle et al, 2020 | Retrospective multicenter matched case–control study on the risk factors for intussusception in infants less than 1 year of age with a special focus on rotavirus vaccines – the German Intussusception Study | Germany | Case-control study | - | 388 |
| 111 | Le Vu et al, 2022 | Age and sex-specific risks of myocarditis and pericarditis following Covid-19 messenger RNA vaccines | France | SCCS study | Case-control study | 35475 |
| 112 | Wan et al, 2022 | Herpes zoster related hospitalization after inactivated (CoronaVac) and mRNA (BNT162b2) SARS-CoV-2 vaccination: A self-controlled case series and nested case-control study | Hong Kong, China | Case-control study | - | 5498 |

| No. | Author, Year | Title | Area | Primary Design | Secondary Design | Sample Size |
|---|---|---|---|---|---|---|
| 113 | Kleinstern et al, 2020 | Vaccination History and Risk of Lymphoma and Its Major Subtypes | the United States | Case-control study | - | 4714 |
| 114 | Lophatananon et al, 2021 | Shingles, Zostavax vaccination and risk of developing dementia: a nested case–control study—results from the UK Biobank cohort | The United Kingdom | Case-control study | Cohort study | 228223 |
| 115 | Abu-Rumeileh et al, 2021 | Varicella zoster virus-induced neurological disease after COVID-19 vaccination: a retrospective monocentric study | Germany | Case-control study | - | 22 |
| 116 | Geier et al, 2018 | Premature Puberty and Thimerosal-Containing Hepatitis B Vaccination: A Case-Control Study in the Vaccine Safety Datalink | the United States | Case-control study | - | 54684 |
| 117 | Shemer et al, 2021 | Association of COVID-19 Vaccination and Facial Nerve Palsy: A Case-Control Study | Israel | Case-control study | - | 111 |
| 118 | Panagiotakopoulos et al, 2021 | Evaluating the Association of Stillbirths After Maternal Vaccination in the Vaccine Safety Datalink | the United States | Case-control study | Cohort study | 3975 |
| 119 | Wan et al, 2021 | Bell's palsy following vaccination with mRNA (BNT162b2) and inactivated (CoronaVac) SARS-CoV-2 vaccines: a case series and nested case-control study | Hong Kong, China | Case-control study | SCCS study | 1479 |
| 120 | Lai et al, 2022 | Carditis After COVID-19 Vaccination With a Messenger RNA Vaccine and an Inactivated Virus Vaccine | Hong Kong, China | Case-control study | - | 1693 |
| 121 | Palmsten et al, 2022 | Influenza vaccination during pregnancy and risk of selected major structural noncardiac birth defects, National Birth Defects Prevention Study 2006–2011 | the United States | Case-control study | - | 13170 |
| 122 | Murata et al, 2021 | Kawasaki Disease and Vaccination: Prospective Case-Control and Case-Crossover Studies among Infants in Japan | Japan | Case-control study | SCCS study | 241 |
| 123 | Sing et al, 2022 | COVID-19 vaccines and risks of hematological abnormalities: Nested case–control and self-controlled case series study | Hong Kong, China | Case-control study | SCCS study | 81822 |

SCCS Study: Self-Controlled Case Series Study

*Sample sizes not specified in the study were replaced with vaccine doses.

**Supplementary Table 2: Minimum Data Sets**

| Category | Data/variable | No.(%) |
|---|---|---|
| Outcome | Diagnosis | 119(96.7) |
| | Date of diagnosis | 60(48.8) |
| | Examination | 36(29.3) |
| | Treatment setting | 33(26.8) |
| | Treatment outcome | 33(26.8) |
| | Death | 29(23.6) |
| | Date of onset | 13(10.6) |
| | Birth weight | 11(8.9) |
| | Date of admission | 8(6.5) |
| | Mode of delivery | 8(6.5) |
| | Date of death | 8(6.5) |
| | Date of discharge | 7(5.7) |
| Demographic data | Geographic information | 110(89.4) |
| | Age | 100(81.3) |
| | Sex | 99(80.5) |
| | Race/Ethnicity | 54(43.9) |
| | Birth date | 30(24.4) |
| | Socio-economic status | 29(23.6) |
| | Occupation | 20(16.3) |
| | Individual identifier | 19(15.4) |
| | Macro indicators | 19(15.4) |
| | Educational status | 13(10.6) |
| | Marital status | 8(6.5) |
| Vaccine | Vaccine name | 109(88.6) |
| | Date of vaccination | 102(82.9) |
| | Vaccine type | 76(61.8) |
| | Dose | 63(51.2) |
| | Technical route | 50(40.7) |
| | Manufacturer | 47(38.2) |
| | Vaccine potency | 23(18.7) |
| | Lot number | 12(9.8) |
| | Route administration | 11(8.9) |
| | Adjuvant | 11(8.9) |
| | Vaccine presentation | 9(7.3) |
| | Strain | 8(6.5) |
| | Date of approval | 7(5.7) |
| Covariate | Diagnosis | 102(82.9) |
| | History | 52(42.3) |
| | Examination | 48(39) |
| | Drugs | 48(39) |
| | Smoking | 41(33.3) |

| **Category** | **Data/variable** | **No.(%)** |
|---|---|---|
| | Body mass index | 27(22.0) |
| | Parity | 24(19.5) |
| | Place of care | 24(19.5) |
| | Treatment | 23(18.7) |
| | Treatment setting | 22(17.9) |
| | Relative's information | 20(16.3) |
| | Date of admission | 18(14.6) |
| | Date of medication | 18(14.6) |
| | Date of diagnosis | 18(14.6) |
| | Birth weight | 18(14.6) |
| | Date of last menstrual period | 16(13) |
| | Date of delivery | 15(12.2) |
| | Alcohol use | 15(12.2) |
| | Date of pregnancy | 12(9.8) |
| | Healthcare utilisation | 12(9.8) |
| | Date of antenatal care | 11(8.9) |
| | Number of visits to the doctor | 10(8.1) |
| | Urban/Rural | 10(8.1) |
| | Date of discharge | 9(7.3) |
| | Insurance | 9(7.3) |
| | Age at delivery | 9(7.3) |
| | Mother's educational status | 9(7.3) |
| | Mother's race/ethnicity | 8(6.5) |
| | Death | 8(6.5) |
| | Weight | 7(5.7) |
| | Date of examination | 7(5.7) |
| | Age of mother | 7(5.7) |

**Supplementary Table 3: Minimum Data Sets for COVID-19 vaccines**

| Category | Data/variable | No.(%) |
|---|---|---|
| Outcome | Diagnosis | 47(95.9) |
| | Date of diagnosis | 27(55.1) |
| | Treatment setting | 20(40.8) |
| | Examination | 16(32.7) |
| | Death | 16(32.7) |
| | Treatment outcome | 14(28.6) |
| | Birth weight | 6(12.2) |
| | Mode of delivery | 5(10.2) |
| | Date of death | 6(12.2) |
| | Date of onset | 5(10.2) |
| | Date of discharge | 4(8.2) |
| | Date of admission | 4(8.2) |
| | Absence | 3(6.1) |
| | Date of examination | 3(6.1) |
| Demographic data | Age | 46(93.9) |
| | Sex | 44(89.8) |
| | Geographic information | 42(85.7) |
| | Race/Ethnicity | 21(42.9) |
| | Occupation | 11(22.4) |
| | Socio-economic status | 10(20.4) |
| | Individual identifier | 6(12.2) |
| | Macro indicators | 5(10.2) |
| | Educational status | 5(10.2) |
| | Birth date | 4(8.2) |
| Vaccine | Vaccine type | 43(87.8) |
| | Date of vaccination | 42(85.7) |
| | Dose | 41(83.7) |
| | Vaccine name | 38(77.6) |
| | Technical route | 30(61.2) |
| | Manufacturer | 29(59.2) |
| | Route administration | 3(6.1) |
| | Lot number | 3(6.1) |
| | Vaccine presentation | 3(6.1) |
| Covariate | Diagnosis | 41(83.7) |
| | Examination | 30(61.2) |
| | Drugs | 22(44.9) |
| | Smoking | 17(34.7) |
| | History | 12(24.5) |
| | Body mass index | 12(24.5) |
| | Date of admission | 12(24.5) |
| | Date of diagnosis | 10(20.4) |

| Category | Data/variable | No.(%) |
|---|---|---|
| | Place of care | 9(18.4) |
| | Treatment setting | 9(18.4) |
| | Treatment | 9(18.4) |
| | Date of medication | 8(16.3) |
| | Parity | 7(14.3) |
| | Date of delivery | 6(12.2) |
| | Date of discharge | 5(10.2) |
| | Date of examination | 5(10.2) |
| | Nursing home resident | 4(8.2) |
| | Death | 4(8.2) |
| | Mode of conception | 3(6.1) |
| | Healthcare utilization | 3(6.1) |
| | Date of consultation | 3(6.1) |

**Supplementary Table 4: Minimum Data Sets for Pregnant Women**

| Category | Data/variable | No.(%) |
|---|---|---|
| Outcome | Diagnosis | 18(100) |
| | Birth weight | 9(50) |
| | Mode of delivery | 8(44.4) |
| | Treatment setting | 6(33.3) |
| | Examination | 5(27.8) |
| | Treatment outcome | 5(27.8) |
| | Length at birth | 2(11.1) |
| | Date of diagnosis | 2(11.1) |
| | Head circumference at birth | 1(5.6) |
| | Temperature | 1(5.6) |
| | Blood pressure | 1(5.6) |
| | Date of discharge | 1(5.6) |
| | Date of onset | 1(5.6) |
| | Date of delivery | 1(5.6) |
| | Date of pregnancy | 1(5.6) |
| | Date of admission | 1(5.6) |
| | Date of fetal death | 1(5.6) |
| | Time of fetal death | 1(5.6) |
| | Date of examination | 1(5.6) |
| | Death | 1(5.6) |
| Demographic data | Geographic information | 16(88.9) |
| | Age | 13(72.2) |
| | Race/Ethnicity | 11(61.1) |
| | Socio-economic status | 7(38.9) |
| | Occupation | 7(38.9) |
| | Marital status | 5(27.8) |
| | Macro indicators | 4(22.2) |
| | Educational status | 4(22.2) |
| | Sex | 3(16.7) |
| | Birth date | 1(5.6) |
| | Name | 1(5.6) |
| Vaccine | Date of vaccination | 16(88.9) |
| | Vaccine name | 16(88.9) |
| | Dose | 8(44.4) |
| | Vaccine type | 8(44.4) |
| | Technical route | 7(38.9) |
| | Manufacturer | 5(27.8) |
| | Vaccine potency | 3(16.7) |
| | Strain | 2(11.1) |
| | Adjuvant | 1(5.6) |
| | Lot number | 1(5.6) |

| Category | Data/variable | No.(%) |
|---|---|---|
| | Vaccine presentation | 1(5.6) |
| Covariate | Diagnosis | 18(100) |
| | Smoking | 15(83.3) |
| | History | 13(72.2) |
| | Date of last menstrual period | 11(61.1) |
| | Parity | 10(55.6) |
| | Body mass index | 10(55.6) |
| | Date of delivery | 10(55.6) |
| | Date of pregnancy | 8(44.4) |
| | Alcohol use | 7(38.9) |
| | Examination | 8(44.4) |
| | Gravidity | 6(33.3) |
| | Date of antenatal care | 5(27.8) |
| | Place of care | 5(27.8) |
| | Treatment setting | 5(27.8) |
| | Birth weight | 4(22.2) |
| | Drug | 4(22.2) |
| | Healthcare utilisation | 4(22.2) |
| | Age at delivery | 3(16.7) |
| | Number of antenatal care visits | 3(16.7) |
| | Treatment | 3(16.7) |
| | Sexual behavior | 3(16.7) |
| | Insurance | 2(11.1) |
| | Mode of delivery | 2(11.1) |
| | Scale | 2(11.1) |
| | Mother's educational status | 2(11.1) |
| | Mode of conception | 2(11.1) |
| | Drug use | 2(11.1) |
| | Date of diagnosis | 2(11.1) |
| | Height | 2(11.1) |
| | Weight | 2(11.1) |
| | Place of recruitment | 2(11.1) |
| | Number of people residing in the household | 2(11.1) |
| | Distance of the home to the hospital | 1(5.6) |
| | Level of influenza activity | 1(5.6) |
| | Day care | 1(5.6) |
| | Biological sample | 1(5.6) |
| | Fertility | 1(5.6) |
| | Infant feeding style | 1(5.6) |
| | Sex of infant | 1(5.6) |
| | Menstrual cycle | 1(5.6) |
| | Main caregiver | 1(5.6) |

| **Category** | **Data/variable** | **No.(%)** |
|---|---|---|
| | Maternal residential province | 1(5.6) |
| | Contraceptive methods | 1(5.6) |
| | Health indicators | 1(5.6) |
| | Multivitamin and mineral supplements use | 1(5.6) |
| | Drug abuse | 1(5.6) |
| | Smoking during pregnancy | 1(5.6) |
| | Drinking alcohol during pregnancy | 1(5.6) |
| | Exercise | 1(5.6) |
| | Date of examination | 1(5.6) |
| | Date of admission | 1(5.6) |
| | Date of medication | 1(5.6) |
| | Age of mother | 1(5.6) |
| | Maternal age at conception | 1(5.6) |

**Supplementary Table 5: All variables collected**

| Category | Data/variable | No.(%) |
|---|---|---|
| Outcome | Diagnosis | 119(96.7) |
| | Date of diagnosis | 60(48.8) |
| | Examination | 36(29.3) |
| | Treatment setting | 33(26.8) |
| | Treatment outcome | 33(26.8) |
| | Death | 29(23.6) |
| | Date of onset | 13(10.6) |
| | Birth weight | 11(8.9) |
| | Date of admission | 10(8.1) |
| | Mode of delivery | 8(6.5) |
| | Date of death | 8(6.5) |
| | Date of discharge | 8(6.5) |
| | Biological sample | 4(3.3) |
| | Absence | 3(2.4) |
| | Length at birth | 2(1.6) |
| | Temperature | 2(1.6) |
| | Date of medication | 2(1.6) |
| | Scale | 2(1.6) |
| | Age of onset | 1(0.8) |
| | Age at diagnosis | 1(0.8) |
| | Head circumference at birth | 1(0.8) |
| | Blood pressure | 1(0.8) |
| | Date of delivery | 1(0.8) |
| | Date of consultation | 1(0.8) |
| | Date of pregnancy | 1(0.8) |
| | Date of examination | 1(0.8) |
| | Date of claim | 1(0.8) |
| | Date of fetal death | 1(0.8) |
| | Time of fetal death | 1(0.8) |
| | Time of diagnosis | 1(0.8) |
| | Duration of symptoms | 1(0.8) |
| | Date of symptom control | 1(0.8) |
| | Date of worst symptoms | 1(0.8) |
| | Duration of hospitalization | 1(0.8) |
| | Date of transfer | 1(0.8) |
| | Date of referral | 1(0.8) |
| | Neuropsychological evaluation | 1(0.8) |
| | Place of care | 1(0.8) |
| | Healthcare utilization | 1(0.8) |
| | Healthcare provider information | 1(0.8) |
| Demographic data | Geographic information | 110(89.4) |

| Category | Data/variable | No.(%) |
| --- | --- | --- |
| | Age | 100(81.3) |
| | Sex | 99(80.5) |
| | Race/Ethnicity | 54(43.9) |
| | Birth date | 30(24.4) |
| | Socio-economic status | 29(23.6) |
| | Occupation | 20(16.3) |
| | Individual identifier | 19(15.4) |
| | Macro indicators | 19(15.4) |
| | Educational status | 13(10.6) |
| | Marital status | 8(6.5) |
| | Name | 4(3.3) |
| Vaccine | Vaccine name | 109(88.6) |
| | Date of vaccination | 102(82.9) |
| | Vaccine type | 76(61.8) |
| | Dose | 63(51.2) |
| | Technical route | 50(40.7) |
| | Manufacturer | 47(38.2) |
| | Vaccine potency | 23(18.7) |
| | Lot number | 12(9.8) |
| | Route administration | 11(8.9) |
| | Adjuvant | 11(8.9) |
| | Vaccine presentation | 9(7.3) |
| | Strain | 8(6.5) |
| | Date of approval | 7(5.7) |
| | Site of vaccination | 3(2.4) |
| | Place of vaccination | 1(0.8) |
| | Healthcare provider information | 1(0.8) |
| Covariate | Diagnosis | 102(82.9) |
| | History | 52(42.3) |
| | Examination | 48(39) |
| | Drugs | 48(39) |
| | Smoking | 41(33.3) |
| | Body mass index | 27(22) |
| | Parity | 24(19.5) |
| | Place of care | 24(19.5) |
| | Treatment | 23(18.7) |
| | Treatment setting | 22(17.9) |
| | Relative's information | 20(16.3) |
| | Date of admission | 18(14.6) |
| | Date of medication | 18(14.6) |
| | Date of diagnosis | 18(14.6) |
| | Birth weight | 18(14.6) |

| Category | Data/variable | No.(%) |
|---|---|---|
| | Date of last menstrual period | 16(13) |
| | Date of delivery | 16(13) |
| | Alcohol use | 15(12.2) |
| | Date of pregnancy | 12(9.8) |
| | Healthcare utilisation | 12(9.8) |
| | Date of antenatal care | 11(8.9) |
| | Number of visits to the doctor | 10(8.1) |
| | Urban/Rural | 10(8.1) |
| | Date of discharge | 9(7.3) |
| | Insurance | 9(7.3) |
| | Age at delivery | 9(7.3) |
| | Mother's educational status | 9(7.3) |
| | Mother's race/ethnicity | 8(6.5) |
| | Death | 8(6.5) |
| | Weight | 7(5.7) |
| | Date of examination | 7(5.7) |
| | Age of mother | 7(5.7) |
| | Number of antenatal care visits | 6(4.9) |
| | Mode of conception | 6(4.9) |
| | Gravidity | 6(4.9) |
| | Smoking during pregnancy | 6(4.9) |
| | Infant feeding style | 6(4.9) |
| | Health indicators | 5(4.1) |
| | Concomitant vaccine | 5(4.1) |
| | Nursing home resident | 5(4.1) |
| | Scale | 5(4.1) |
| | Mode of delivery | 4(3.3) |
| | Level of influenza activity | 4(3.3) |
| | Language | 4(3.3) |
| | Mother's body mass index | 4(3.3) |
| | Height | 4(3.3) |
| | Time of hospital visit | 4(3.3) |
| | Date of surgery | 4(3.3) |
| | Place of recruitment | 4(3.3) |
| | Sexual behavior | 4(3.3) |
| | Biological sample | 3(2.4) |
| | Fertility | 3(2.4) |
| | Immigration | 3(2.4) |
| | Birth country | 3(2.4) |
| | Date of onset | 3(2.4) |
| | Exercise | 3(2.4) |
| | Drug use | 2(1.6) |

| Category | Data/variable | No.(%) |
|---|---|---|
| | Drinking alcohol during pregnancy | 2(1.6) |
| | Contraceptive methods | 2(1.6) |
| | Date of death | 2(1.6) |
| | Date of Immigration | 2(1.6) |
| | Date of treatment | 2(1.6) |
| | Day care | 2(1.6) |
| | Type of admission | 2(1.6) |
| | Vital status | 2(1.6) |
| | Cause of death | 2(1.6) |
| | Psychological factors | 2(1.6) |
| | Healthcare provider information | 2(1.6) |
| | Genetic information | 2(1.6) |
| | Menstrual cycle | 2(1.6) |
| | Maternal age at conception | 2(1.6) |
| | Number of people residing in the household | 2(1.6) |
| | Folate intake | 2(1.6) |
| | Length at birth | 1(0.8) |
| | Vital status | 1(0.8) |
| | Temperature | 1(0.8) |
| | Head circumference | 1(0.8) |
| | Virome data | 1(0.8) |
| | Birth order | 1(0.8) |
| | Housing style | 1(0.8) |
| | Hg exposure | 1(0.8) |
| | Distance of the home to the hospital | 1(0.8) |
| | Type of family | 1(0.8) |
| | Civil status | 1(0.8) |
| | Day care | 1(0.8) |
| | Social support | 1(0.8) |
| | Sex of infant | 1(0.8) |
| | Transplant type | 1(0.8) |
| | Susceptibility for infections | 1(0.8) |
| | Medical conditions | 1(0.8) |
| | Main caregiver | 1(0.8) |
| | Religion | 1(0.8) |
| | Maternal country of birth | 1(0.8) |
| | Maternal residential province | 1(0.8) |
| | Age of menarche | 1(0.8) |
| | Hours/week of work | 1(0.8) |
| | Rotating shift work | 1(0.8) |
| | Sun exposure | 1(0.8) |
| | Sleep duration | 1(0.8) |

| Category | Data/variable | No.(%) |
|---|---|---|
| | Multivitamin and mineral supplements use | 1(0.8) |
| | Drug abuse | 1(0.8) |
| | Prenatal vitamin use | 1(0.8) |
| | Night shift work | 1(0.8) |
| | Pet ownership during pregnancy | 1(0.8) |
| | Date of enrollment | 1(0.8) |
| | Date of transplant | 1(0.8) |
| | Time of admission | 1(0.8) |
